# How We Choose One over Another: Predicting Trial-by-Trial Preference Decision

**DOI:** 10.1371/journal.pone.0043351

**Published:** 2012-08-17

**Authors:** Vidya Bhushan, Goutam Saha, Job Lindsen, Shinsuke Shimojo, Joydeep Bhattacharya

**Affiliations:** 1 Department of Electronics & Electrical Communication Engineering, Indian Institute of Technology, Kharagpur, India; 2 Department of Psychology, Goldsmiths, University of London, London, United Kingdom; 3 Division of Biology, California Institute of Technology, Pasadena, California, United States of America; University College London, United Kingdom

## Abstract

Preference formation is a complex problem as it is subjective, involves emotion, is led by implicit processes, and changes depending on the context even within the same individual. Thus, scientific attempts to predict preference are challenging, yet quite important for basic understanding of human decision making mechanisms, but prediction in a group-average sense has only a limited significance. In this study, we predicted preferential decisions on a trial by trial basis based on brain responses occurring before the individuals made their decisions explicit. Participants made a binary preference decision of approachability based on faces while their electrophysiological responses were recorded. An artificial neural network based pattern-classifier was used with time-frequency resolved patterns of a functional connectivity measure as features for the classifier. We were able to predict preference decisions with a mean accuracy of 74.3±2.79% at participant-independent level and of 91.4±3.8% at participant-dependent level. Further, we revealed a causal role of the first impression on final decision and demonstrated the temporal trajectory of preference decision formation.

## Introduction

Unable to choose between a bale of hay and a pail of water, Buridan’s hungry and thirsty ass, when placed in the middle of two options, starved to death as a victim of its inability to make a rational decision of choosing one over the other. But we, human being, are ‘born to choose’, and the desire to make choices, even at the cost of rationality, is crucial for our well-being [1]. For example, most consumers, when asked to choose one out of two identical food samples express a preference for one, rather than choosing a no preference option [2]; this effect is found to be robust against personal expectations, explicit instructions and personal traits [3].

Preference is a fundamental component of the processes of internal evaluation of choices or alternatives which underlies general decision making [4]. Preference is one of the most challenging topics in the research field of decision making, partly because it is subjective (i.e. there is no externally defined correct answer and it can vary across individuals), involves emotion, led by implicit processes, and changes depending on the context within the same individual (i.e. a decision in one trial may differ from another). Therefore, scientific attempts to predict preference are crucial for basic understanding of decision making, as well as for real-world applications. Further, preference decisions are often made intuitively without explicit reasoning and almost instinctively. Thus, prediction at a group-averaged level has only a limited significance, and therefore, it is important to predict preference decision on trial-by-trial basis based on implicit measures. A recent attempt has been made to predict subjective preference for drinks, out of two possible choices, from single-trial brain responses [5] but is fraught with difficulties [6], [7]; the achieved average prediction accuracy (53.57%) was only marginally better than chance (50%). This highlights the important challenge in decoding preference decisions from implicit brain responses on trial-by-trial basis.

Faces play a very important role in our social life, and we make complex social decisions, from mate-selection in our lives to candidate-selection in a political election, based on mere facial appearance and/or attractiveness [8]. Extensive research has been made to identify a set of facial features which make a face attractive [9]. Possibly no research is needed to predict which face a heterosexual male would prefer when asked to choose between Megan Fox (voted as one of the most desirable women) and Jocelyn Wildenstein (voted as one of the ugliest celebrities).

But we know little about how one makes a preference decision when the two faces are closely matched (e.g., age, race, gender, gaze, facial attributes, facial emotion), i.e. under decision conflict [10]. Does first impression contribute to the final preference decision? Are decision patterns specific to individual or common across individuals? What is the temporal trajectory of the formation of a preference decision? Most importantly, can one predict such preference decision under conflict on trial-by-trial basis even before the individuals make their decisions explicit?

In the current study, we addressed these challenging mind-reading problems by applying machine learning techniques [11] to electrical brain responses recorded from human participants while they were making preference decisions, based on approachability of faces, in a two-alternative forced choice task paradigm.

## Materials and Methods

### Participants

Eighteen adult healthy human participants (14 women, age range of 18–35 years) took part in the study. They received a fixed amount of cash (£ 20) for their participation. All participants gave their written informed consent before the beginning of the experiment. Experimental protocols, set according to the Helsinki declaration, were approved by the Local Ethics Committee of the Department of Psychology, Goldsmiths, University of London.

### Stimuli

The stimuli were human faces which were generated by a computer software (www.facegen.com), which was used in previous studies on face perception [12], [13]. All faces were emotionally neutral, and contained no hair. Faces were subsequently paired in terms of gender, race, age, and independently obtained ratings of approachability. The approachability construct is regularly used in the literature concerning social judgements [14]–[17]. For example, Adolphs et al. [18] argue in favouring this construct because (1) it is a clear measure of real-life social judgement, (2) it is easy to understand, and (3) it has a relative low variability across participants.

### Procedure

The participants performed a simple preference decision task in which they had to choose, from a pair of two closely matched faces, the face that they would most like to approach and to talk to. Each trial started with a fixation cross presented for 500 ms, followed by the onset of a face. The participants viewed this first face as long as they wanted. When the participants pressed a key, the first face was replaced by another fixation cross for 500 ms and followed by a second face. Within a trial, the first and second faces were closely matched as mentioned earlier. There was also no restriction on the viewing time of the second face. However, the participants were instructed to make a response as soon as they reached a decision, and they indicated their preferred face by making a left or right hand response (counterbalanced across participants) corresponding to the two faces (see Figure S1 for a trial outline). Each participant performed 39 trials.

### Data Acquisition and Pre-processing

EEG signals were recorded from 64 electrodes by using a BioSemi ActiveTwo^(R)^ amplifier. The vertical and horizontal eye movements were recorded by four additional electrodes. The sampling frequency was 512 Hz. The signals were filtered with a 3rd order sinc low pass filter with a −3 dB cut-off at approximately 128 Hz. EEG signals were referenced to the average of two mastoid electrodes. Trials with artefacts were discarded after visual inspection, and eye-blinks artefacts were corrected by ICA based EEGLAB [19]. EEG data from one participant was removed due to excessive artefacts; however, behavioural data from all participants were used in statistical analysis.

### Data Analysis

In terms of decisions, there are two possibilities: the first face chosen or the second face chosen. As we were interested in early components only, we considered the first 1 s period starting from the onset of each face for our analysis, and termed them as F1X for the first face, and F2X for the second face. Note that explicit decisions were made a considerable time after F2X, so these brain responses during F2X supposedly reflect the implicit components of preference decision formation. In this study classification for F1X and F2X was done separately as F1C-F1NC and F2C-F2NC. If the first face (F1) was chosen at the end of a trial then the specific trial was termed as F2NC (face-2 Not Chosen). Otherwise if face-2 was chosen, the trial was termed as F2C (Face-2 Chosen). Correspondingly goes for F1C and F1NC.

For the classification of neural signals, we needed to extract those features which would capture the neuronal mechanisms underlying the preference decision. Synchronization between near and distant brain regions is often considered as the substrate for complex cognitive tasks including decision making [20], [21]. Since synchronization could be frequency-specific [22], [23], we computed the Time-Frequency resolved Synchronization Likelihood (TFSL) [24], which is a modified concept of synchronization likelihood (SL) [25]. SL is based on the concept of generalized synchronization [26], and is sensitive to both linear and non-linear couplings. Briefly, the calculation of TFSL involves five steps as follows:

- Definition of the frequency band of interest followed by band-pass filtering.
- Construction of time-delay embedding vectors that represent dynamical states of the underlying multidimensional system supposedly generating the signals under study.
- Localization of the times of recurrent dynamical states in both systems.
- Computation of the synchronization likelihood (SL) that the recurrence of a state in one system is accompanied by a recurrent state in the other system.
- Repetition of steps (iii) and (iv) at different time points in order to obtain a time series of SL values.

EEG data were filtered using 10th order Butterworth band-pass filter for six frequency bands: delta (1–4 Hz), theta (4–8 Hz), alpha (8–12 Hz), beta (12–30 Hz), gamma-1 (30–40 Hz) and gamma-2 (40–60 Hz). TFSL was calculated in these six frequency bands for all electrode pairs. The synchronization likelihood (SL) parameters which are common across frequency bands are (the symbols have their usual meaning followed in [24]: *P*_ref_ = 0.1, shift in state embedded vector = 1, shift in reference embedded vector = 25, and number of reference embedded vectors = 20. Other SL parameters are specific to individual frequency bands, and they are shown in the Table 1.

**Table 1:** The synchronization likelihood parameters for six frequency bands which were used for calculating the *TFSL*.

Embedding Parameters	Delta	Theta	Alpha	Beta	Gamma-1	Gamma-2
*L* (lag of embedding vector)*m* (dimension of embedding vector)	258	217	146	68	55	36

TFSL at each frequency band was calculated for every 20 intervals of 50 ms each after the onset of the face (both for F1X and F2X). Features are named as **B** *_i_* **E** *_j_* **T** *_k_* where 1≤ *i* ≤6; 1≤ *j* ≤64; 1≤ *k* ≤20; and *i* corresponds to each of 6 frequency **B**ands, *j* correspond to each of 64 **E**lectrodes and *k* corresponds to each of 20 **T**ime intervals. Hence the total number of features become [freq band (6) × electrode (64) × time windows (20)] = 7680 for one trial of one participant.

From this a set of features was selected which were used in classification by using artificial neural network (ANN) [27] based classifier. Two-layered feed-forward back-propagating ANN with 16 neurons in hidden layer and 4 neurons in output level was used for the analysis. Tan-Sigmoid Transfer Function was used in hidden layer while Linear Transfer function in output layer. The neural network was trained using a Levenberg-Marquardt back-propagation algorithm. Maximum number of epochs was set as 100 and the performance goal (MSE) as 10e−5.

In order to keep the computational cost low and to reduce the redundancy among the features, features were shortlisted based on their ability to classify the pattern and their ranks. The ranking of features was done using *F*-Ratio [28], which is defined as the ratio of variance of means between the classes (two classes: “face chosen (FC)” and “face not chosen (FNC)”) and average variance within the classes for the same feature.

The optimum number of features was selected by the Sequential forward selection (SFS) method [11], which is a popular feature selection method to reduce the dimension of the extracted features. The SFS consists of the following steps. (i) Calculate the *F*-ratio for all the features and rank them in descending order. (ii) Train the ANN based classifier (as described afterwards) to differential between the trial cases only with rank-1 feature; let the classification accuracy achieved from test data with this selected feature be *C_k_*_ = 1_%. (iii) Repeat the step 2 with rank-1 and rank-2 features. Let the classification accuracy be *C_k_*_ = 2_% which is greater or equal to *C_k_*_ = 1_%. (iii) Continue adding lower ranked features and repeat the earlier steps till the classification accuracy does not increase with increasing number of features. In this way we get different classification accuracy as *C_k_*_ = 1_%, *C_k_*_ = 2_%, …, *C_k_*_ = *n*_%. (v) From the above steps we get the final set of features (total number of features  = *n*) that optimally represents the data set and the associated classification accuracy is *C_k_*_ = *n*_%.

For each subject, 60% of the trials were randomly selected and used for training the feed-forward back-propagation type network with 2 layers. Another set of 20% was used for validating the trained network. Predictability of the network was tested on the remaining 20% of the data and accuracy was recorded. The whole procedure was repeated 10 times with a different set of training, validation and test data. The final accuracy was averaged across these ten runs.

In order to strengthen the notion of unbiased estimation, *k*-fold cross validation technique [29] (for *k* = 5 and 10) was also used for comparing the results (for results see Table S2). The classification accuracy achieved through these methods varies within ±1%.

The analysis was made on two models: (i) combined global model (CGM), reflecting participant-independent factors where data of all the participants were pooled together, and (ii) personalized average model (PAM) where data from each participants were treated individually reflecting participant-dependent factors (see Ref. [30] for a similar attempt on evaluating within-participant or participant-dependent and cross-participant or participant-independent classifiers). In CGM analysis, all trials across participants were analyzed together in a single classifier. However, in PAM analysis, trial cases of individual participants were analyzed separately in individual classifiers.

Next, we studied the time profile of the feature discrimination characteristics as expressed by *F*-ratios, and performed two broad types of analysis. The first one used increasing length of data segment from the face onset (i.e. evaluating the gradual decision process as the information is accumulated) and consists of the following steps. (i) Calculate *TFSL* for time window 0–50 ms (*k* = 1 where *k* is the number of time points). (ii) Calculate *F*-ratio, which is a measure of discrimination ability (*FR_ijk_*, as in Ref. [28]) for each feature **B** *_i_* **E** *_j_* **T** *_k_* (total number of features for 0–50 ms time window is 64×6×1 = 384). (iii) Rank the *F*-ratio in descending order. (iv) Take the average *F*-ratio of top 300 features. (v) Repeat steps (i)–(iv) for longer time windows in steps of 50 ms, i.e., 0−k*50 ms (*k* = 2, 3, …, 20) and estimate the temporal profile of *F*-ratio. The second analysis was similar to the above except we analysed data in non-onverlapping 50 ms time windows, 0–50 ms, 50–100 ms, 100–150 ms, …, 950–1000 ms.

## Results and Discussion

While analysing the behavioural data, we observed that sequential presentation of faces does not lead to any order related preference: the mean likelihood of first face being chosen (0.51±0.08) did not differ significantly from the chance level, 0.5 (one-sample *t*-test: *t*(17) = 0.43, *p* = 0.673, n.s.). Neither any systematic preference towards any particular face was observed nor the average image of all preferred faces was very similar to the average image of all non-preferred faces (see Figure S2). Further, the subjective exposure to faces did not influence preference formation as the mean viewing time for preferred faces (1518±260 ms) was not significantly different (paired *t*-test: *t*(17) = 0.42, *p* = 0.681, n.s.) from the mean viewing time for non-preferred faces (1535±260 ms). Based on these results, it could be suggested that the order effect (i.e. presentation sequence) and the exposure effect (i.e. the viewing time for individual faces) did not significantly influence preference decisions as studied here.

**Figure 1:**
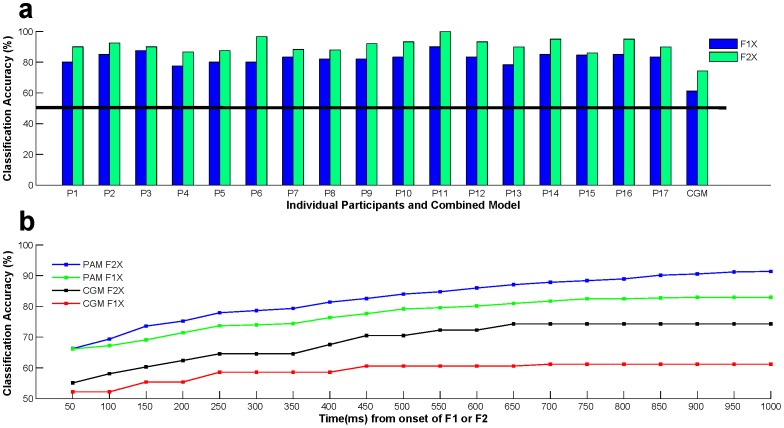
Classification accuracy at individual and combined level. (**a**) Prediction performance of the classifier at individual level (PAM; P*i* indicates *i*-th Participant) and at combined level (CGM). Blue and green bars represent classifier accuracy based on brain responses related to the first face (F1X) and second face (F2X), respectively. Prediction accuracy was higher at individual level than at combined level, and also higher for F2X than F1X. Chance level is at 50% (black horizontal line). (b) Classifier performance for PAM and CGM analysis for both F1X and F2X with respect to time. The classifier performance gradually increased till 700 ms especially for CGM.

In the classification analysis, first, we applied our classification technique using the first 1 s of brain responses from the onset of second faces (F2X). The average classifier accuracy for CGM was 74.3±2.79%, which was higher than chance level of 50% in a binary decision task. This CGM accuracy reflects the common decision making pattern (global tendency towards decision) across all participants. Interestingly, the average classifier accuracy for PAM analysis rose to 91.39±3.8% (Fig. 1a). Though there are some variations in the classifier performance among the participants (range: 86–100%), the overall accuracy is significantly higher (one sample *t*-test: *t*(16) = 15.75, *p*<.001) than the chance-performance (50%) of PAM classifier, suggesting that idiosyncratic mechanisms of preference decision making were successfully captured by our classifier. This was also reflected by the fact that the number of features that optimally predict individual’s preference decisions widely varied across participants (See Table S1). These distinctions between CGM and PAM models are not too surprising as sharp differences were also reported between within- and cross-participant classifiers of reward related fMRI responses [30].

Next we asked whether our classifier could predict the decision based on initial (1 s) brain responses to first face only (F1X). Note that the second face was not explored yet, so any prediction based on these brain responses would indicate the first impression effect [31]. The prediction accuracy for CGM was 61.2±2.94%, (Fig. 1a), still above the chance level at 50%, but lower than the accuracy for F2X as expected, since most of the decision processes were supposed to be made after the onset of second face. The average prediction accuracy for PAM was considerably high at 82.94±3.21% (range of 80–90%, see Fig. 1a and Table S1), suggesting systematic idiosyncratic mechanisms of formation of first impression. This is consistent with fMRI study by Kim et al. (2007) on the first impression of facial attractiveness. Note that prediction accuracy was systematically higher for F2X than for F1X, i.e. TFSL obtained from EEG activity has higher predictive power after presentation of the second face as compared to the first face. neural responses to the second face had higher predictive power.

We further investigated the time profile of preference decision formation. The classifier performance was evaluated by gradually incorporating longer time windows from the onset of two faces (see *Methods* for details). The classifier performance for CGM for F1X increased monotonically till 450 ms and afterwards reached a plateau of 60% (Fig. 1b). This suggests that the first impression process common across participants occurred within half a second of the presentation of a facial stimulus. On the other hand, the classifier performance for PAM for F1X reached a plateau around 700 ms, showing the temporal trajectory of first impression formation process within participants. The classifier performance for CGM for F2X also reached a plateau at 700 ms, but performance for participant dependent model for F2X kept increasing till the end of analysed period. This suggests that participants deliberated over the second faces a bit longer than the first faces. Note that the classifier performance was better for F2X than for F1X for the entire duration, as expected.

**Figure 2:**
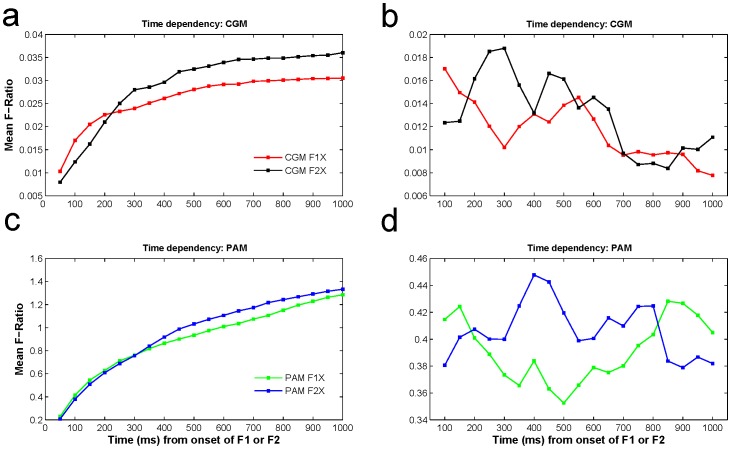
Time dependency of average *F*-ratio distribution at individual and combined level. (**a**) Temporal profiles of average *F*-ratio for data available (from the face onset) till that time for CGM for F1X (in red) and for F2X (in black). (**b**) Temporal profiles of average *F*-ratio for successive 50 ms time window for CGM for F1X (in red) and for F2X (in black). (**c**) Temporal profiles of average *F*-ratio for data available (from the face onset) till that time for PAM for F1X (in green) and for F2X (in blue). (**d**) Temporal profiles of average *F*-ratio for successive 50 ms time window for PAM for F1X (in green) and for F2X (in blue).

We also studied the separation in feature space at different time windows in terms of averaged *F*-ratio over top 300 features (See *Methods*). First we calculated *F*-ratio over increasing time period started from the face onset, which would indicate the accumulated progression of separability in feature space. We observed that the discrimination ability was considerably better for F2X than for F1X for time periods later than 200 ms (Fig. 2a). Next we calculated *F*-ratio at sequential non-overlapping time windows, which would indicate the separation specificity of any time window in feature space. The average *F*-ratio was highest during time window 250–300 ms for F2X (CGM model) while it was relatively low for F1X over the same time window (Fig. 2b). Almost a similar trend is observed for PAM model (Fig. 2d) during 400–500 ms. Consistent with our earlier result (Fig. 1b), the feature space for CGM lacked discrimination power after 700 ms indicating saturation, while for PAM it continued to increase till the end of the analysed time window (Fig. 2c).

As there was no clear discernible local maximum in the temporal profiles of the predictive power of TFSL, these results altogether suggest that the preference decision was possibly not made at a specific moment in time that was consistent across trials, but rather spreading over time (or trial) as a process of dynamically evolving bias with discretionary stages in this case.

**Figure 3:**
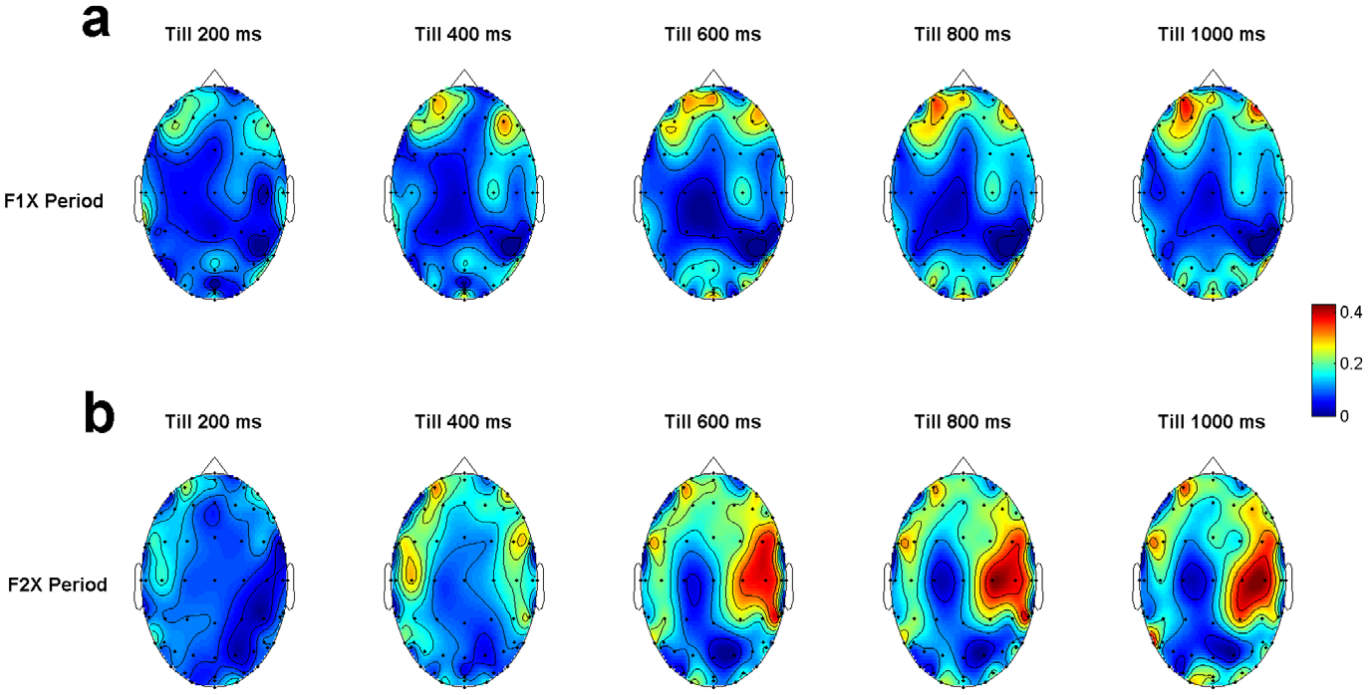
Scalp maps of average *F*-ratio distribution for CGM analysis at different time periods. (**a**), (**b**) Analysis for F1X and F2X, respectively. Note that the frontal electrode region has higher *F*-ratio in case of F1X while right temporal electrode region has higher *F*-ratio for F2X.

We did not find any frequency band showing consistently higher discrimination power, therefore, results were averaged across frequency bands, and the spatial maps of averaged *F*-ratio were shown in Fig. 3 (see Methods S1). For CGM, the frontal electrode regions had higher separation for F1X while right temporal electrode regions had higher separation for F2X. The similar analysis for PAM shows that frontal and left temporal electrode regions showed maximum separation for F1X (see Figure S3). The left frontal and right temporal electrode regions (same as CGM) showed maximum separation for F2X, and for both cases, other electrode regions got involved as time progressed (see Figure S3).

Let us offer a few practical remarks. First, our reported mean accuracy of 74.3% for participant-independent classifier was considerably higher than that (53.25%) reported by Chau and Damouras [7] in a similar task involving subjective preference decision. They recorded near-infrared spectroscopy (NIRS) signals from the prefrontal cortex while the participants were asked to decide which one of the two sequentially presented drinks they preferred and used Fisher’s linear discriminant analysis as the classifier to decode subjective preference. We suggest that a combination of factors including the comprehensive coverage of EEG frequency bands, the adopted measure of functional connectivity, a broader sampling of brain regions (as compared to NIRS), and an excellent temporal resolution of EEG signals have contributed to this much higher prediction accuracy. Second, the chance level of our classifier is set at 50%, which is theoretically the case for a two-alternative choice task. However, actual chance probability does not always converge to theoretical chance probability due to several factors/features in the dataset. Therefore, we further attempted a quasi-randomization procedure by considering 50% correct and 50% incorrect target for the training dataset; the selection of these 50% was made at random and the prediction accuracy was subsequently averaged across runs. The mean (SD) classification chance accuracy for the F1X and F2X was 49.8% (3.2) and 47.8% (3.7), respectively. Therefore, our obtained classification accuracy was indeed found to be higher than at chance level. Third, some of the *F*-maps (Fig. 3a) are similar in distribution to eye-movements, but our results are unlikely due to ocular artefacts as we found, on close inspection, that the frontal effects in these *F*-maps were not produced by the low frequency oscillatory components, the major component of eye movements. Further, note that we did not observe any frequency band showing consistently higher discrimination power, therefore, results were averaged across frequency bands. Fourth, since the mapping of response hand to preference for either face 1 or face 2 was counterbalanced across participants, the high classification accuracy as observed by the individualized PAM analysis might partly reflect decoding of preparatory motor responses rather than decoding decision processes related to face preference. However, the distinction between the onset of an intention to respond and the moment of decision is rather diffuse, and we believe that decoding preparatory motor responses does not fully explain our accuracy in predicting preference decisions. Regarding our analysis of F1C vs F1NC, the motor response occurring at the end of F1 was not related to the final decision made after viewing F2. Although the participants were free to press either with the left or the right hand to progress to viewing F2, 15 out of 18 participants consistently used one hand to do so. For the participants that did use both hands at the end of F1, the correlation between the choice of key after F1 and F2 did not exceed ±0.22. However, this still leaves the possibility that our analyses of F1C vs F1NC might decode a planned subsequent response to F2. Further, planning of an one-handed response is characterized by contra-lateralized neuronal activity patterns with distinct signatures on time, electrode or brain regions, and EEG frequency domains [32]–[35]. If decoding of preparatory motor responses was driving the prediction accuracies as reported in this study, maximum discriminatory power should be observed in these lateralized patterns [36], yet such specificity was clearly not observed in our studied feature spaces (see earlier). Nevertheless, one could not rule out a contribution of planned motor responses, especially at the later stages of studied epochs that were temporally close (i.e. within ∼1 s) to the response; future research on predicting decision should aim first to separate the decision related responses from motor related ones. It is important here to note that since response mapping to preference was counterbalanced over participants this issue is mostly relevant for the individualized PAM results but not for the group-based CGM results. Finally, instead of adopting regions of interest-based prediction techniques, which are often used in fMRI-based brain decoding [30], [37]–[39], we adopted a mechanistic machine learning approach where one searches for the best set of features which yield the best classification in a validating dataset. We treated sites of brain activity and the nature of brain oscillations agnostically - that is, without any reference to prior hypotheses. Our primary assumption was that, regardless of how an individual’s brain represents the information relevant for preference decision, it does so consistently. The representations may be dispersed over space, time, frequency, network patterns, and also over individuals, but they could still be reliably detected through the machine learning techniques. Because such data driven analysis techniques are not reliant on the activation patterns of a small subset of brain regions or frequency band, they have substantially increased sensitivity to detect the patterns specific to decisions. Further, such techniques involve statistical associations of complex activation patterns that occur when an individual preference decision is being made, “it does not depend on the vagaries of an experimenter interpreting the meaning of an activation map” [40]. Although this mechanistic approach limits the scope of neurophysiological interpretations, it amplifies the possibility of being adapted for real-world applications of brain decoding.

In summary, we presented the mind-reading result of a complex social judgement task. Earlier mind-reading evidence was related to tasks with predominant sensory components [16], [38], [41] or based on activations of pre-selected brain regions [42]–[44]. Here, we showed that it is possible to predict subjective decision of approachability of faces with high accuracy based on synchronization between multiple brain regions without any prior hypothesis. The classification process is entirely adaptive and data-driven. Our results also identified idiosyncratic and common brain responses of preference decision. Finally, the analysed brain responses were most likely implicit and pre-conscious, yet we showed that they possessed significant ability to predict explicit preference decision. Altogether, we suggest that our proposed approach of trial-by-trial prediction (with relatively small dataset from a particular individual), together with the high range of predictability, offers promising potential as real-world applications such as neuromarketing, social networking, and neural lie detection.

## Supporting Information

Figure S1 **Timing sequence of each trial.** F1/2 and RT1/2 indicate onset and reaction times of the first/second face, respectively. The reaction (viewing) times, RT1 and RT2, varied across trials as the viewing time was unrestricted for both faces. Note that the explicit decision of each trial was made after viewing the second face.
(TIF)

Figure S2 **Mean preferred (left) and non-preferred (right) face.**
(TIF)

Figure S3 **Scalp maps of average** ***F*** **-ratio distribution for PAM analysis at different time periods.** (**a**) Analysis for F1X. (**b**) Analysis for F2X. Note that the frontal and left temporal regions have higher average *F*-ratio for F1X while right temporal and left anterior regions have higher average *F*-ratio for F2X.
(TIFF)

Table S1 **Mean classification accuracy and standard deviation (SD) for different models.** Prediction performance of the artificial neural network based classifier at user-dependent level (personalized average model, PAM) and at user-independent level (CGM). First column represents the participant number; second column represents the number of features selected after implementing sequential feature selection method; third column represents the prediction accuracy (in percentage) by analysing brain responses to first face (F1X); fourth column represents the prediction accuracy (in percentage) by analysing responses to second face (F2X). The first and last row represents the mean prediction accuracy and standard deviation for CGM and averaged PAM, respectively. The Standard deviation for CGM model and individual participants are calculated across repetitions while the standard deviation of PAM model is across participants.
(DOC)

Table S2 **The classification accuracy for different models.**
(DOC)

Methods S1 **Spatial map profile analysis based on** ***F*** **-ratio.**
(DOC)
