# Supplementary figures and images for: How We Choose One over Another: Predicting Trial-by-Trial Preference Decision
Source: PLoS One. 2012 Aug 17;7(8):e43351. doi: 10.1371/journal.pone.0043351 (PMC3422291; doi:10.1371/journal.pone.0043351)

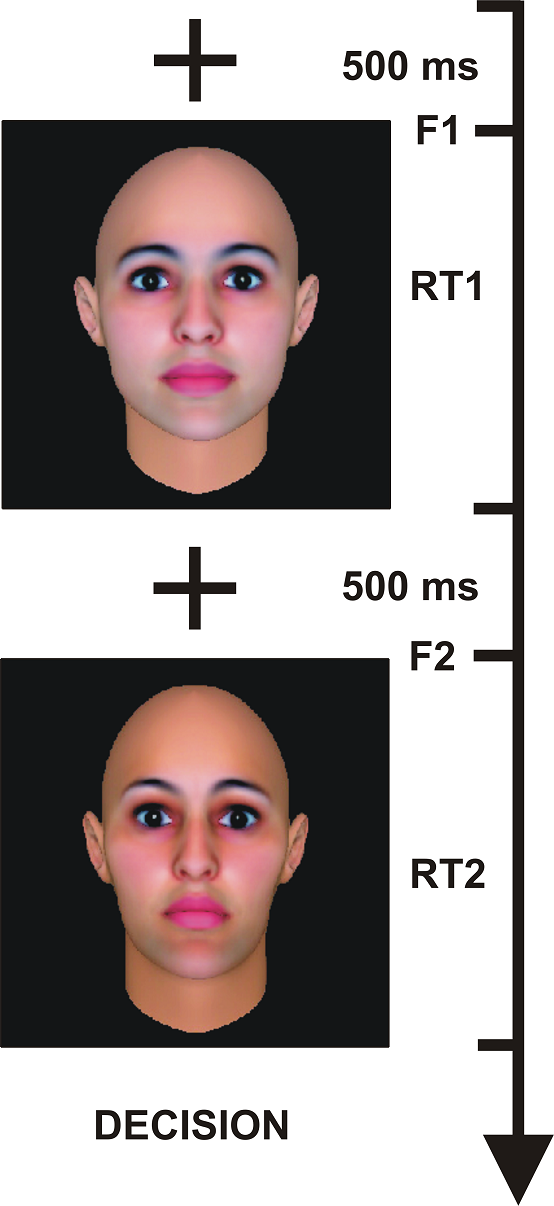

Supplement: Figure S1 — Timing sequence of each trial. F1/2 and RT1/2 indicate onset and reaction times of the first/second face, respectively. The reaction (viewing) times, RT1 and RT2, varied across trials as the viewing time was unrestricted for both faces. Note that the explicit decision of each trial was made after viewing the second face. (TIF) [file pone.0043351.s001.tif]

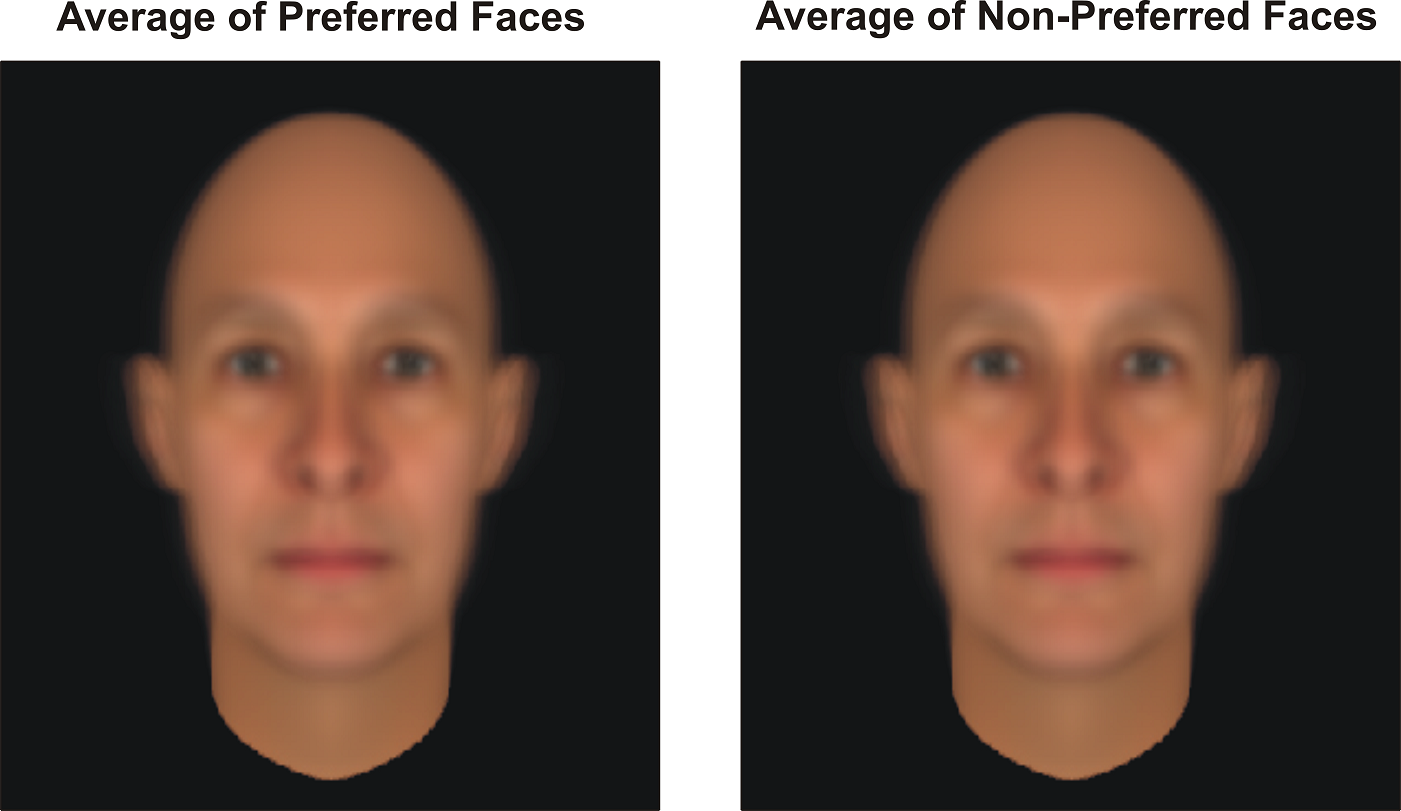

Supplement: Figure S2 — Mean preferred (left) and non-preferred (right) face. (TIF) [file pone.0043351.s002.tif]

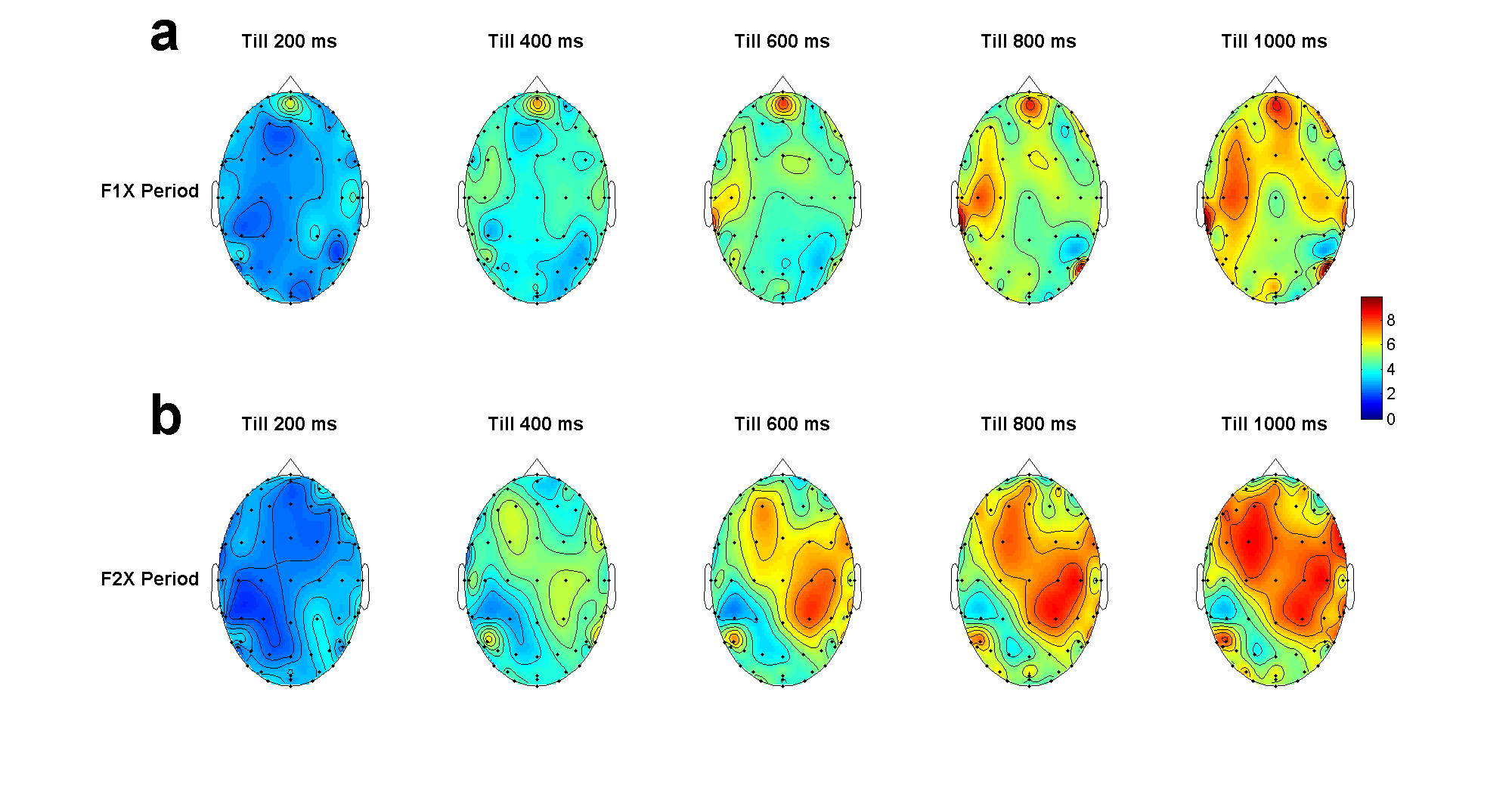

Supplement: Figure S3 — Scalp maps of average F -ratio distribution for PAM analysis at different time periods. (a) Analysis for F1X. (b) Analysis for F2X. Note that the frontal and left temporal regions have higher average F-ratio for F1X while right temporal and left anterior regions have higher average F-ratio for F2X. (TIFF) [file pone.0043351.s003.tiff]
