# Supplementary material for: How We Choose One over Another: Predicting Trial-by-Trial Preference Decision
Source: PLoS One. 2012 Aug 17;7(8):e43351. doi: 10.1371/journal.pone.0043351 (PMC3422291; doi:10.1371/journal.pone.0043351)
Supplement: Table S1 — Mean classification accuracy and standard deviation (SD) for different models. Prediction performance of the artificial neural network based classifier at user-dependent level (personalized average model, PAM) and at user-independent level (CGM). First column represents the participant number; second column represents the number of features selected after implementing sequential feature selection method; third column represents the prediction accuracy (in percentage) by analysing brain responses to first face (F1X); fourth column represents the prediction accuracy (in percentage) by analysing responses to second face (F2X). The first and last row represents the mean prediction accuracy and standard deviation for CGM and averaged PAM, respectively. The Standard deviation for CGM model and individual participants are calculated across repetitions while the standard deviation of PAM model is across participants. (DOC) [file pone.0043351.s004.doc]

| **Participant Number** | | **No of Features** | **% Accuracy**  **F1X** | **% Accuracy**  **F2X** |
| --- | --- | --- | --- | --- |
|  |  | |  |  |
| CGM | 5-40 | | **61.2 ± 2.94** | **74.3 ± 2.79** |
|  |  | |  |  |
| 1 | 5-20 | | 80±8.7 | 90±6.4 |
| 2 | 40-100 | | 85±7.8 | 92.5±4.8 |
| 3 | 10-20 | | 87.5±7.8 | 90±5.6 |
| 4 | 175-200 | | 77.5±9.7 | 86.6±8.9 |
| 5 | 30-40 | | 80±8.8 | 87.5±7.8 |
| 6 | 30-40 | | 80±9.2 | 96.6±3.2 |
| 7 | 300-400 | | 83.3±7.8 | 88.3±7.9 |
| 8 | 300-350 | | 82±9.4 | 88±7.26 |
| 9 | 100-300 | | 82±8.4 | 92±4.8 |
| 10 | 100-200 | | 83.3±9.2 | 93.2±5.1 |
| 11 | 10-20 | | 90±6.8 | 99.5±1.5 |
| 12 | 100-300 | | 83.3±8.3 | 93.2±5.3 |
| 13 | 50-100 | | 78.3±9.3 | 89.9±7.8 |
| 14 | 20-100 | | 85±7.9 | 95±4.6 |
| 15 | 60-70 | | 84.6±8.1 | 86±8.7 |
| 16 | 10-100 | | 85±9.2 | 95±4.2 |
| 17 | 30-100 | | 83.3±8.9 | 89.9±8.7 |
| PAM |  | | **82.94±3.21** | **91.39±3.80** |

**Table S1. Mean classification accuracy and standard deviation (SD) for different models.** Prediction performance of the artificial neural network based classifier at user-dependent level (personalized average model, PAM) and at user-independent level (CGM). First column represents the participant number; second column represents the number of features selected after implementing sequential feature selection method; third column represents the prediction accuracy (in percentage) by analysing brain responses to first face (F1X); fourth column represents the prediction accuracy (in percentage) by analysing responses to second face (F2X). The first and last row represents the mean prediction accuracy and standard deviation for CGM and averaged PAM, respectively. The Standard deviation for CGM model and individual participants are calculated across repetitions while the standard deviation of PAM model is across participants.
