# Supplementary material for: How We Choose One over Another: Predicting Trial-by-Trial Preference Decision
Source: PLoS One. 2012 Aug 17;7(8):e43351. doi: 10.1371/journal.pone.0043351 (PMC3422291; doi:10.1371/journal.pone.0043351)
Supplement: Table S2 — The classification accuracy for different models. (DOC) [file pone.0043351.s005.doc]

| **CGM** | **% Classification accuracy  (Randomised training)** | | **% Classification accuracy**  **(*k*-Fold Cross validation)** | |
| --- | --- | --- | --- | --- |
|  | *N* = 10 | *N* = 50 | *k* = 5 | *k* = 10 |
| F1X | 61.2±2.94 | 61.3±2.65 | 60.8±2.89 | 60.4±3.47 |
| F2X | 74.3±2.79 | 73.8±2.67 | 73.6±3.23 | 73.8±3.84 |

**Table S2.** The classification accuracy for différent models.
