# Supplementary material for: How We Choose One over Another: Predicting Trial-by-Trial Preference Decision
Source: PLoS One. 2012 Aug 17;7(8):e43351. doi: 10.1371/journal.pone.0043351 (PMC3422291; doi:10.1371/journal.pone.0043351)
Supplement: Methods S1 — Spatial map profile analysis based on F -ratio. (DOC) [file pone.0043351.s006.doc]

**Supplementary Methods:**

**Spatial map profile analysis based on F-Ratio**

- **Steps for average *F*-Ratio for each channel with data available till that time for CGM**

Step 1: TFSL was calculated for time window 0-200 ms (*k*=4 where *k* is the number of time points)

Step 2: Corresponding *F*-ratio (*FR_ijk_*) for each feature **B***_i_***E***_j_***T***_k_* were calculated (total feature for 0-200ms is 64*6*4=1536)

Step 3: They were sorted according to the value of *FR_ijk_*. Feature with highest *F*-ratio is given Rank-1.

Step 4: F-ratio corresponding to each channel in top 300 were added separately and divided by sum of top 300 *FR_ijk_.* This step gives the average F-ratio corresponding to each channel till above mentioned time window.

Step 5: Step 1 to 4 was repeated for below time windows:

0-400 ms (*k*=8), 0-800 ms (*k*=16), 0-1000 ms (*k*=20)

Step 6 : Results are plotted as scalp maps ( Fig. 3a for F1X and Fig. 3b for F2X)

- **Steps for average *F*-Ratio for each channel with data available till that time for PAM**

Here all the steps for both the above methods were same except that entire process is repeated for each participant and then their scores were averaged to get the results (Fig. S3a and Fig. S3b).
